# Supplementary material for: Dynamic modes of Notch transcription hubs conferring memory and stochastic activation revealed by live imaging the co-activator Mastermind
Source: eLife. 2024 May 10;12:RP92083. doi: 10.7554/eLife.92083 (PMC11087053; doi:10.7554/eLife.92083)
Supplement: Supplementary file 1. — Summary of Drosophila strains (Table 2). Genetic combinations for each figure (Table 3). p-values from statistical tests (Table 4). [file elife-92083-supp1.docx]

**De Haro Arbona *et al*: Supplementary File 1.**

Supplementary File 1—Table 1: Genomic co-ordinates and oligonucleotides used for CRISPR, constructs and qPCR.

Supplementary File 1—Table 2: Summary of Drosophila strains.

Supplementary File 1—Table 3: Genetic combinations for each figure

Supplementary File 1—Table 4: p-values from statistical tests**.**

**Supplementary File 1—Table 1: Genomic co-ordinates and oligonucleotides used for CRISPR, constructs and qPCR.**

| Mam gRNA | ATGAATATTTGGGTAAGATT |
| --- | --- |
| Mam homology arm upstream | 2R:14009456..14010278 |
| Mam homology arm downstream | 2R:14009456..14010278 |
| Med1 gRNA | TAGGAACGCATAGATATGAG |
| Med1 homology arm upstream | 3L:21633297..21634295 |
| Med1 homology arm downstream | 3L:21634297..21635295 |
| *E(spl)mβ HLH* gRNA | GGGACGCCACATGGGGCCAG |
| *E(spl)mβ HLH* homology arm upstream | 3R:26005336..26006118 |
| *E(spl)mβ-HLH* homology arm downstream | 3R:26004521..26005328 |
| CSL nt IDR | 2L:15039938..15040106 |
| CSL ct IDR | 2L:15042401..15042618 |
| NICD IDR | X: 3170337..3170534 |
| NICD PEST IDR | X: 3169065..3170534 |
| Med1 IDR | 3L:21629087..21632060 |
| Mam IDR | 2R:14055747..14057930 |
| Mam nIDR | 2R:14026225..14064332 |
| cDNA: RPL32 fwd | ATGCTAAGCTGTCGCACAAATG |
| cDNA: RPL32 rvs | GTTCGATCCGTAACCGATGT |
| cDNA: Hairless fwd | CATCGCTGAGCTTTTCGGAC |
| cDNA: Hairless rvs | CTCGGCCAAGCGTACTGTTC |
| cDNA: CBP/p300 fwd | AACGCCATACCAGGCATGAA |
| cDNA: CBP/p300 rvs | GAATTGATCATCCCACCGCC |
| cDNA: Med13 fwd | TGGAAGTGGTGTCGGGTGGT |
| cDNA: Med13 rvs | GCCAGAACAGCTGACTCGGC |
| cDNA: Cdk8 fwd | AGATGAAAACGCAGATAGAGCG |
| cDNA: Cdk8 rvs | TTCTTTGCCATCGCTTGTCT |

**Supplementary File 1—Table 2: Summary of Drosophila strains.**

| Short name | Notes | Source | RRID, reference. |
| --- | --- | --- | --- |
| 1151-Gal4 |  |  | FBti0007229 |
| vas-phiC31;;M [attP86Fb] | Used for 3^rd^ chromosome insertions | Bloomington Stock Center | RRID:BDSC_24749 |
| UAS-N∆ECD | 2^nd^ and 3^rd^ chromosome insertions (unknown and attP86Fb insertion) | ^37,38^ |  |
| eGFP::CSL | Genomic fragment insertion attP86Fb (RRID:BDSC 24749) | ^34^ |  |
| Halo::CSL | Genomic fragment insertion attP86Fb | ^69^ |  |
| vas-phiC31;;M [attP51D] | Used for 2^nd^ chromosome insertions |  | RRID:BDSC 24483 |
| eGFP::Hairless | Genomic fragment insertion attP51D | ^34^ |  |
| Hairless::Halo | Genomic fragment insertion attP51D | ^42^ |  |
| Med13::YFP |  | Bloomington Stock Center | RRID:BDSC_57899 |
| UAS-ParA1-mCherry | Insertion attP86Fb | ^34^ |  |
| UAS-ParB1-GFP | Insertion attP86Fb | ^34^ |  |
| *E(spl)-C[*m∂ intA] | CRISPR of E(spl)-C | ^34^ |  |
| UAS-Hairless-RNAi |  | Bloomington Stock Center; Line used in: ^34^ | RRID:BDSC_27315 |
| UAS-Mam-RNAi |  | Bloomington Stock Center; Line used in: ^34,104,105^ | RRID:BDSC_28046 |
| UAS-Med13-RNAi |  | Bloomington Stock Center; Line used in: ^106,107^ | RRID:BDSC_34630 |
| UAS-nejire-RNAi |  | Vienna Resource Center | KK10288 |
| UAS-Cdk8-RNAi |  | ^61^ |  |
| UAS-Mam[DN] |  | ^67^ |  |
| MS1096-Gal4 |  | Bloomington Stock Center | RRID:BDSC_8860 |
| nanos-phiC31; Msp300[attP40] | Used for 2^nd^ chromosome insertions of UAS-IDR | Bloomington Stock Center | RRID:BDSC_25709 |
| UAS-Cry2-TevC | Insertion attP51C (RRID:BDSC 24482) | ^69^ |  |
| UAS-OptIC-Notch{ω}[mCherry] | Insertion attP40 | ^69^ |  |
| *E(spl)-mβ*-HLH-MS2-LacZ | CRISPR of E(spl)-*mβ* | This paper, modified from ^11^ |  |
| hsp83-MCP::GFP |  | Bloomington Stock Center | RRID:BDSC_7280 |
| nos-cas9 |  | Bloomington Stock Center | RRID:BDSC_54591 |

| αtub-piggyBac |  | Bloomington Stock Center | RRID:BDSC_32070 |
| --- | --- | --- | --- |
| sfGFP::Mam | CRISPR of Mam locus | This paper |  |
| Halo::Mam | CRISPR of Mam locus | This paper |  |
|  |  |  |  |
|  |  |  |  |
| H2AV::Halo |  | ^42^ |  |
| sfGFP::Med1 | CRISPR of Med1 locus | This paper |  |
| eGFP::Rbp3 | CRISPR of Rbp3 locus | ^16^ |  |
| mCherry::Rbp1 | CRISPR of Rbp1 locus | ^16^ |  |
| UAS-sfGFP-NICD[IDR] | Insertion attp40 | This paper |  |
| UAS-sfGFP-NICD[IDRPEST] | Insertion attp40 | This paper |  |
| UAS-sfGFP-CSL[ntIDR] | Insertion attp40 | This paper |  |
| UAS-sfGFP-CSL[ctIDR] | Insertion attp40 | This paper |  |
| UAS-sfGFP-CSL[ctIDR] | Insertion attp40 | This paper |  |
| UAS-sfGFP-Med1[IDR] | Insertion attp40 | This paper |  |
| UAS-sfGFP-Mam[nIDR] | Insertion attp40 | This paper |  |
| UAS-sfGFP-Mam[IDR] | Insertion attp40 | This paper |  |

**Supplementary File 1—Table 3: Genetic combinations used for each Figure**

| **Figure** | **X chromosome** | **II chromosome** | **III chromosome** |  |
| --- | --- | --- | --- | --- |
| Figure 1B | *1151-Gal4* | *UAS-N∆ECD, GFP::Mam* | *E(spl)-C[m∂ intA}, UAS-ParA::mCherry* |  |
| Figure 1B | *1151-Gal4* | *UAS-N∆ECD* | *GFP::CSL, E(spl)-C[m∂ intA}, UAS-ParA::mCherry* |  |
| Figure 1C | *1151-Gal4* | *UAS-N∆ECD, GFP::Mam* | *UAS-white RNAi* |  |
| Figure 1C | *1151-Gal4* | *UAS-N∆ECD* | *UAS-white RNA, GFP::CSL* |  |
| Figure 1C | *1151-Gal4* | *UAS-N∆ECD, GFP::Mam* | *UAS-Hairless RNAi* |  |
| Figure 1C | *1151-Gal4* | *UAS-N∆ECD, GFP::Hairless* | *E(spl)-C[m∂ intA}, UAS-ParA::mCherry* |  |
| Figure 1C’ | *1151-Gal4* | *UAS-N∆ECD* | *UAS-Hairless RNAi, GFP::CSL* |  |
| Figure 1C’ | *1151-Gal4* | *UAS-N∆ECD* | *UAS-white-RNAi, GFP::CSL* |  |
| Figure 1D | *1151-Gal4* | *UAS-LacZ* | *E(spl)-C[m∂ intA}, UAS-ParB::GFP, Halo::CSL* |  |
| Figure 1D | *1151-Gal4* | *UAS-N∆ECD* | *E(spl)-C[m∂ intA}, UAS-ParB::GFP, Halo::CSL* |  |
| Figure 1D | *1151-Gal4* | *UAS-LacZ, Halo::Mam* | *E(spl)-C[m∂ intA}, UAS-ParB::GFP* |  |
| Figure 1D | *1151-Gal4* | *UAS-N∆ECD, Halo::Mam* | *E(spl)-C[m∂ intA}, UAS-ParB::GFP* |  |
| Figure 1E-E’ | *1151-Gal4* | *UAS-N∆ECD* | *E(spl)-C[m∂ intA}, UAS-ParB::GFP, Halo::CSL* |  |
| Figure 1E-E’ | *1151-Gal4* | *UAS-N∆ECD, Halo::Mam* | *E(spl)-C[m∂ intA}, UAS-ParB::GFP* |  |
| Figure 1E-E’ | *1151-Gal4* | *UAS-N∆ECD, Halo ::Hairless* | *E(spl)-C[m∂ intA}, UAS-ParA::mCherry* |  |
| Figure S1C | *ms1096-Gal4, tub-Gal80ts* | *UAS-N∆ECD, GFP::Mam* | *mCherry::CSL* |  |
| Figure S1D | *1151-Gal4* | *UAS-N∆ECD* | *UAS-white RNAi* |  |
| Figure S1D | *1151-Gal4* | *UAS-N∆ECD* | *UAS-Hairless RNAi* |  |
| Figure S1E | *As Figure 1C* | | |  |
| Figure S1F | *As Figure 1D-E’* | | |  |
| Figure 2A | *1151-Gal4* | *UAS-N∆ECD* | *GFP::CSL, 12xCSLsites* |  |
| Figure 2A | *1151-Gal4* | *UAS-N∆ECD* | *GFP::CSL, 48xCSLsites* |  |
| Figure 2C | *ms1096-Gal4, tub-Gal80ts* | *UAS-N∆ECD, UAS-NICD[PEST IDR]::GFP* | *E(spl)-C[m∂ intA}, UAS-ParA::mCherry* |  |
| Figure 2C | *ms1096-Gal4, tub-Gal80ts* | *UAS-N∆ECD, UAS-CSL[ntIDR]::GFP* | *E(spl)-C[m∂ intA}, UAS-ParA::mCherry* |  |
| Figure 2D | *1151-Gal4* | *UAS-N∆ECD, GFP::Mam* | *E(spl)-C[m∂ intA}, UAS-ParA::mCherry* |  |
| Figure 2D | *1151-Gal4* | *UAS-N∆ECDΔIDR, GFP::Mam* | *E(spl)-C[m∂ int A}, UAS-ParA::mCherry* |  |
| Figure 2E | *1151-Gal4* | *UAS-N∆ECD, Halo::Mam* | *E(spl)-C[m∂ intA}, UAS-ParB::GFP* |  |
| Figure 2E | *1151-Gal4* | *UAS-N∆ECD* | *E(spl)-C[m∂ intA}, UAS-ParB::GFP, Halo::CSL* |  |
| Figure S2A | *1151-Gal4* | *UAS-N∆ECD* | *E(spl)-C[m∂ intA}, UAS-ParA::mCherry, GFP::CSL* |  |
| Figure S2A | *1151-Gal4* | *UAS-N∆ECD, GFP::Mam* | *E(spl)-C[m∂ intA}, UAS-ParA::mCherry* |  |
| Figure S2B | *ms1096-Gal4, tub-Gal80ts* | *UAS-N∆ECD, UAS-Mam[nIDR ]::GFP* | *E(spl)-C[m∂ intA}, UAS-ParA::mCherry* |  |
| Figure S2C | *ms1096-Gal4, tub-Gal80ts* | *UAS-N∆ECD, UAS-CSL[ctIDR]:: GFP* | *E(spl)-C[m∂ intA}, UAS-ParA::mCherry* |  |
| Figure S2C | *ms1096-Gal4, tub-Gal80ts* | *UAS-N∆ECD, UAS-NICD[IDR]:: GFP* | *E(spl)-C[m∂ intA}, UAS-ParA::mCherry* |  |
| Figure S2C | *ms1096-Gal4, tub-Gal80ts* | *UAS-N∆ECD, UAS-Med1[IDR]-GFP* | *E(spl)-C[m∂ intA}, UAS-ParA::mCherry* |  |
| Figure S2C | *ms1096-Gal4, tub-Gal80ts* | *UAS-N∆ECD, UAS-Mam[IDR]-GFP* | *E(spl)-C[m∂ intA}, UAS-ParA::mCherry* |  |
| Figure S2C | *ms1096-Gal4, tub-Gal80ts* | *UAS-N∆ECD, UAS-Mam[nIDR]-GFP* | *E(spl)-C[m∂ intA}, UAS-ParA::mCherry* |  |
| Figure 3A-C | *1151-Gal4* | *UAS-N∆ECD, GFP::Mam* | *E(spl)-C[m∂ intA}, UAS-ParA::mCherry* |  |
| Figure 3D | *1151-Gal4* | *GFP::Mam, UAS-yellow-RNAi* | *UAS-N∆ECD* |  |
| Figure 3D | *1151-Gal4* | *GFP::Mam, UAS-nej-RNAi* | *UAS-N∆ECD* |  |
| Figure 3E | *1151-Gal4* | *UAS-N∆ECD, GFP::Mam* | *UAS-white-RNAi* |  |
| Figure 3E | *1151-Gal4* | *UAS-N∆ECD, GFP::Mam* | *UAS-med13-RNAi* |  |
| Figure 3E | *1151-Gal4* | *UAS-N∆ECD* | *UAS-white-RNAi, GFP::CSL* |  |
| Figure 3E | *1151-Gal4* | *UAS-N∆ECD* | *UAS-med13-RNAi, GFP::CSL* |  |
| Figure 3E | *1151-Gal4* | *UAS-N∆ECD, GFP::Mam* | *UAS-med13-RNAi* |  |
| Figure 3F | *1151-Gal4* | *UAS-N∆ECD, GFP::Mam* | *E(spl)-C[m∂ intA}, UAS-ParA::mCherry* |  |
| Figure 3F | *1151-Gal4* | *UAS-N∆ECD* | *E(spl)-C[m∂ intA}, UAS-ParA::mCherry, GFP::CSL* |  |
| Figure 3G | *1151-Gal4* | *UAS-N∆ECD, GFP::Mam* | *UAS-white-RNAi* |  |
| Figure 3G | *1151-Gal4* | *UAS-N∆ECD, GFP::Mam* | *UAS-cdk8-RNAi* |  |
| Figure 3H | *1151-Gal4* | *UAS-N∆ECD* | *E(spl)-C[m∂ intA}, UAS-ParA::mCherry, skd(Med13)::YFP* |  |
| Figure 3H | *1151-Gal4* | *UAS-LacZ* | *E(spl)-C[m∂ intA}, UAS-ParA::mCherry, skd(Med13)::YFP* |  |
| Figure S3A | *1151-Gal4* | *UAS-N∆ECD, GFP::Mam* | *E(spl)-C[m∂ intA}, UAS-ParA::mCherry* |  |
| Figure S3A-D | *1151-Gal4* | *UAS-N∆ECD, GFP::Mam* | *E(spl)-C[m∂ intA}, UAS-ParA::mCherry* |  |
| Figure S3E | *1151-Gal4* | *UAS-N∆ECD, GFP::Mam* | *UAS-white-RNAi* |  |
| Figure S3E | *1151-Gal4* | *UAS-N∆ECD, GFP::Mam* | *UAS-med13-RNAi* |  |
| Figure S3E | *1151-Gal4* | *UAS-N∆ECD, GFP::Mam* | *UAS-white-RNAi* |  |
| Figure S3F | *1151-Gal4* | *UAS-N∆ECD, GFP::Hairless* | *E(spl)-C[m∂ intA}, UAS-ParA::mCherry* |  |
| Figure S3G | *1151-Gal4* | *UAS-N∆ECD, mCherry::CSL[LLL]* | *E(spl)-C[m∂ intA}, UAS-ParA::GFP* |  |
| Figure S3H, I | *1151-Gal4* | *UAS-N∆ECD, GFP::Mam* | *E(spl)-C[m∂ intA}, UAS-ParA::mCherry* |  |
| Figure S3J | *1151-Gal4* | *UAS-N∆ECD* | *UAS-white-RNAi* |  |
| Figure S3J | *1151-Gal4* | *UAS-N∆ECD, UAS CBP/p300 RNAi* |  |  |
| Figure S3J | *1151-Gal4* | *UAS-N∆ECD* | *UAS-med13-RNAi* |  |
| Figure S3J | *1151-Gal4* | *UAS-N∆ECD* | *UAS-cdk8-RNAi* |  |
| Figure 4A | *1151-Gal4* | *GFP::Mam, UAS-yellow-RNAi* | *UAS-N∆ECD* |  |
| Figure 4A | *1151-Gal4* | *GFP::Mam, UAS-Mam[DN]* | *UAS-N∆ECD* |  |
| Figure 4A' | *1151-Gal4* | *UAS-N∆ECD, UAS-LacZ* | *GFP::CSL* |  |
| Figure 4A' | *1151-Gal4* | *UAS-N∆ECD, UAS-Mam[DN]* | *GFP::CSL* |  |
| Figure 4B | *1151-Gal4* | *UAS LacZ, UAS-LacZ* |  |  |
| Figure 4B | *1151-Gal4* | *UAS-N∆ECD, UAS-LacZ* |  |  |
| Figure 4B | *1151-Gal4* | *UAS LacZ, UAS-Mam[DN]* |  |  |
| Figure 4B | *1151-Gal4* | *UAS-N∆ECD, UAS-Mam[DN]* |  |  |
| Figure 4C | *1151-Gal4* | *UAS-N∆ECD, UAS LacZ* | *E(spl)-C[m∂ intA}, UAS-ParA::mCherry, skd::YFP* |  |
| Figure 4C | *1151-Gal4* | *UAS-N∆ECD, UAS-Mam[DN]* | *E(spl)-C[m∂ intA}, UAS-ParA::mCherry, skd::YFP* |  |
| Figure 4D-D' | *1151-Gal4* | *UAS-N∆ECD, UAS-LacZ* |  |  |
| Figure 4D-D' | *1151-Gal4* | *UAS LacZ, UAS-Mam[DN]* |  |  |
| Figure 4D-D' | *1151-Gal4* | *UAS-N∆ECD, UAS-Mam[DN]* |  |  |
| Figure S4A | *1151-Gal4* | *UAS-N∆ECD, UAS-yellow-RNAi* | *GFP::CSL* |  |
| Figure S4A | *1151-Gal4* | *UAS-N∆ECD, UAS-Mam-RNAi* | *GFP::CSL* |  |
| Figure S4B | *ms1096-Gal4, tub-Gal80ts* | *UAS-NICD[∆IDR-PEST], UAS-LacZ* | *E(spl)-C[m∂ intA}, UAS-ParA::mCherry, UAS-N∆ECD* |  |
| Figure S4B | *ms1096-Gal4, tub-Gal80ts* | *UAS-NICD[∆IDR-PEST], UAS-Mam[DN]* | *E(spl)-C[m∂ intA}, UAS-ParA::mCherry, UAS-N∆ECD* |  |
| Figure 5A-B | *ms1096-Gal4, tub-Gal80ts* | *UAS-N∆ECD, GFP::Mam* | *mCherry::CSL,* |  |
| Figure 5C | *ms1096-Gal4, tub-Gal80ts* | *UAS-N∆ECD,* | *GFP::CSL, E(spl)-C[m∂ intA}, UAS-ParA::mCherry* |  |
| Figure 5E, F, H, I | *1151-Gal4* | *UAS-CRYTEVc, UAS-OptIC-Notch{ω}::mCherry, Halo::Mam* | *GFP::CSL* |  |
| Figure S5A | *ms1096-Gal4, tub-Gal80ts* | *UAS-N∆ECD, GFP::Mam* | *mCherry::CSL,* |  |
| Figure 6A | *1151-Gal4* | *UAS-LacZ, GFP::Mam* | *E(spl)-C[m∂ intA}, UAS-ParA::mCherry* |  |
| Figure 6A | *1151-Gal4* | *UAS-N∆ECD, GFP::Mam* | *E(spl)-C[m∂ intA}, UAS-ParA::mCherry* |  |
| Figure 6B | *1151-Gal4* | *UAS LacZ, GFP::Rbp3* | *E(spl)-C[m∂ intA}, UAS-ParA::mCherry* |  |
| Figure 6B | *1151-Gal4* | *UAS-N∆ECD, GFP::Rbp3* | *E(spl)-C[m∂ intA}, UAS-ParA::mCherry* |  |
| Figure 6C | *1151-Gal4* | *UAS-LacZ* | *E(spl)-C[m∂ intA}, UAS-ParA::mCherry, GFP::Med1* |  |
| Figure 6C | *1151-Gal4* | *UAS-N∆ECD* | *E(spl)-C[m∂ intA}, UAS-ParA::mCherry, GFP::Med1* |  |
| Figure 6D | *1151-Gal4* | *UAS-N∆ECD, hsp83-MCP::GFP* | *E(spl)-C[m∂ intA}, UAS-ParA::mCherry, E(spl)mβ-MS2* |  |
| Figure 6E | *1151-Gal4, Rbp1::mCherry* | *UAS-N∆ECD* | *Halo::CSL, GFP::Med1* |  |
| Figure 6F | *1151-Gal4* | *UAS-N∆ECD, GFP::Rbp3* | *E(spl)-C[m∂ intA}, UAS-ParA::mCherry* |  |
| Figure 6F | *1151-Gal4* | *UAS-N∆ECD* | *E(spl)-C[m∂ intA}, UAS-ParA::mCherry, GFP::Med1* |  |
| Figure 6F | *1151-Gal4* | *UAS-LacZ, hsp83-MCP::GFP* | *E(spl)-C[m∂ intA}, UAS-ParA::mCherry, E(spl)mβ-MS2* |  |
| Figure 6F | *1151-Gal4* | *UAS-N∆ECD, hsp83-MCP::GFP* | *E(spl)-C[m∂ intA}, UAS-ParA::mCherry, E(spl)mβ-MS2* |  |
| Figure S6A | *As Figure 6A* | | |  |
| Figure S6B | *As Figure 6B* | | |  |
| Figure S6C | *As Figure 6C* | | |  |
| Figure S6D | *As Figure 6D* | | |  |
| Figure S6E | *As Figure 6E* | | |  |
| Figure S6F-H | *As Figure 6F* | | |  |

**Supplementary File 1—Table 4: p-values from statistical tests.**

| Figure 1B | Mam OFF vs. ON | n=32, 30 | p=1.4e-11 |
| --- | --- | --- | --- |
| Figure 1B | CSL OFF vs. ON | n=45, 28 | p = 1.4e-12 |
| Figure 1D | CSL OFF vs. ON | n=5, 7 | p=5.5e-3, |
| Figure 1D | Mam OFF vs. ON | n=5, 13 | p=2.3e-4 |
| Figure S1B | Mam ON wRi vs. ON HRi | n=25, 27 | p= 0.43 |
| Figure S1C | CSL ON wRi vs. ON HRi | n=38, 31 | p= 9.8e-06 |
| Figure 2A | ectopic array 12 vs. 48 | n=45, 40 | p=0.09 |
| Figure 2A | *E(spl)-C* 12 vs. 48 | n=45, 40 | p=0.75 |
| Figure 2C | NICD-PEST IDR Notch OFF vs. ON | n=62, 67 | p = 8.4e-03 |
| Figure 2C | CSL-nt IDR Notch OFF vs. ON | n=8, 40 | p=0.63 |
| Figure 2D | Nact FL vs. Nact ∆IDR,, | n=58, 55 | p=4.2e-4 |
| Figure S2C | CSL ct IDR Notch OFF vs. ON | n=39, 44 | p=0.69 |
| Figure S2C | NICD IDR Notch OFF vs. ON | n=83, 72 | p=3.4e-04 |
| Figure S2C | Med1 IDR Notch OFF vs. ON | n=32, 25 | p=0.04 |
| Figure S2C | Mam IDR Notch OFF vs. ON | n=18,36 | p=0.25 |
| Figure S2C | Mam nIDR Notch OFF vs. ON | n=12, 29 | p=0.14 |
| Figure 3A | DMSO vs. triptolide | n=49, 36 | p = 0.55 |
| Figure 3C | DMSO vs. A485 | n=47,62 | p=0.45 |
| Figure 3D | yRNAi vs CBP/p300 RNAi | n=26, 31 | p=0.17 |
| Figure 3E | Mam control vs Mam Med13Ri | n=30, 45 | p=9.8e-7 |
| Figure 3E | CSL control vs CSL Med13Ri | n=92, 36 | p=1.1e-4 |
| Figure 3F | Mam DMSO vs Mam Senexin B | n=27, 43 | p=1.2e-4 |
| Figure 3F | CSL DMSO vs CSL Senexin B | n=34, 37 | p=7.5e-5 |
| Figure 3G | ctrl vs. Cdk8 Ri, | n=33, 32 | p=9.4e-4 |
| Figure 3H | Notch OFF vs. ON | n=28, 31 | p=1.7e-10 |
| Figure S3A | DMSO vs. triptolide | n=18, 16 | p= 0.0118 |
| Figure S3A | DMSO vs. triptolide | n=9,8 | p= 0.17 |
| Figure S3B | DMSO vs. A485 | n=20, 6 | p= 2.9e-4 |
| Figure S3C | Cytoplasmic DMSO vs. A485 | n=32, 22 | p= 0.0081 |
| Figure S3C | *E(spl)-C* DMSO vs. A485 | n=13, 6 | p= 0.59 |
| Figure S3D | DMSO vs. Senexin A | n=27, 26 | p=7.5e-04 |
| Figure S3E | Control KD vs. Med13 KD | n=10, 11 | p= 0.004 |
| Figure S3F | DMSO vs. Senexin B | n=31, 28 | p= 0.71 |
| Figure S3G | DMSO vs. Senexin B | n=27, 31 | p= 4.8e-7 |
| Figure S3H | DMSO vs. NVP2 | n=30, 43 | p=0.83 |
| Figure S3I | DMSO vs. NVP2 | n=31, 14 | p=0.035 |
| Figure 4C | OFF vs. ON | n=28, 31 | p=1.7e-10 |
| Figure 4D’ | Cytoplasmic OFF vs. ON. | n=18, 20 | p=1.5e-3 |
| Figure 4D’ | Cytoplasmic ON ctrl vs. ON MamDN | n=20, 22 | p=8.1e-4 |
| Figure 4D’ | Cytoplasmic OFF vs. ON, MamDN | n=18, 22 | p=0.09 |
| Figure S4B | Control vs. MamDN | n=25, 28 | p=0.76 |
| Figure 5A | Mam 24 hrs ON vs 24 hrs ON, 4 hrs OFF | n=28, 19 | p=0.047 |
| Figure 5A | Mam 24 hrs ON, 8 hrs OFF vs 24 hrs ON, 4 hrs OFF | n=19, 21 | p = 8.9e-5 |
| Figure 5A | Mam 24 hrs ON, 8 hrs OFF vs 24 hrs ON, 4 hrs OFF | n=28, 21 | p = 2.5e-8 |
| Figure 5A | CSL 24 hrs ON vs 24 hrs ON, 4 hrs OFF | n=28, 19 | p = 0.10 |
| Figure 5A | CSL 24 hrs ON, 8 hrs OFF vs 24 hrs ON, 4 hrs OFF | n=19, 21 | p = 0.58 |
| Figure 5A | CSL 24 hrs ON, 8 hrs OFF vs 24 hrs ON, 4 hrs OFF | n=28, 21 | p = 0.22 |
| Figure 5C | CSL 24 hrs ON vs 24 hrs OFF | n=28, 21 | p = 0.42 |
| Figure 5C | Cytoplasmic 24 hrs ON vs 24 hrs OFF | n=30, 16 | p = 5.6e-3 |
| Figure 5C | Nuclear 24 hrs ON vs 24 hrs OFF | n=10, 5 | p = 0.14 |
| Figure 5F | Naïve vs. preactivated OFF | n=38, 21 | p = 8.6e-4 |
| Figure 5F | preactivated OFF vs. preactivated ON | n=21, 43 | p = 2.3e-5 |
| Figure 5F | Naïve vs. preactivated ON | n=38, 43 | p = 0.09 |
| Figure 5I | Naïve vs preactivated | n=11, 9 | p=1.7e-3 |
| Figure S5A | cytoplasmic 24 hrs ON vs 24 hrs ON, 4 hrs OFF | n=22, 30 | p = 0.04 |
| Figure S5A | cytoplasmic 24 hrs ON, 8 hrs OFF vs 24 hrs ON, 4 hrs OFF | n=30, 30 | p = 0.93 |
| Figure S5A | cytoplasmic 24 hrs ON, 8 hrs OFF vs 24 hrs ON, 4 hrs OFF | n=22, 30 | p = 0.06 |
| Figure S5A | nuclear 24 hrs ON vs 24 hrs ON, 4 hrs OFF | n=4, 4 | p = 0.49 |
| Figure S5A | nuclear 24 hrs ON, 8 hrs OFF vs 24 hrs ON, 4 hrs OFF | n=4, 4 | p = 0.73 |
| Figure S5A | nuclear 24 hrs ON, 8 hrs OFF vs 24 hrs ON, 4 hrs OFF | n=4, 4 | p = 0.73 |
